# Effectiveness of Nurse-Led Heart Failure Self-Care Education on Health Outcomes of Heart Failure Patients: A Systematic Review and Meta-Analysis

**DOI:** 10.3390/ijerph17186559

**Published:** 2020-09-09

**Authors:** Youn-Jung Son, JiYeon Choi, Hyeon-Ju Lee

**Affiliations:** 1 Red Cross College of Nursing, Chung-Ang University, Seoul 06974, Korea; yjson@cau.ac.kr; 2 College of Nursing, Mo-Im Kim Nursing Research Institute, Yonsei University, Seoul 03722, Korea; jychoi610@yuhs.ac; 3 Department of Nursing, Tongmyoung University, Busan 48520, Korea

**Keywords:** heart failure, self-care, nursing, systematic reviews, meta-analysis

## Abstract

Poor self-care behaviors can lead to an increase in the risk of adverse health outcomes among patients with heart failure. Although a number of studies have investigated the effectiveness of nurse-led self-care education, the evidence regarding the effects of nurse-led intervention in heart failure remains uncertain. This study aimed to evaluate evidence on the effectiveness of nurse-led heart failure self-care education on health outcomes in patients with heart failure. To identify studies testing nurse-led education designed to improve self-care among heart failure patients, comprehensive search methods were used between January 2000 and October 2019 to systematically search six electronic databases: PubMed, CINAHL, Embase, Cochrane library, Web of Science, and SCOPUS. All the eligible study data elements were independently assessed and analyzed using random-effects meta-analysis methods. Of 612 studies, eight articles were eligible for this study. Nurse-led heart failure self-care education significantly reduced the risk of all-cause readmission (risk ratio (RR) = 0.75, 95% confidence interval (CI) = 0.66–0.85), heart failure specific readmission (RR = 0.60, 95% CI = 0.42–0.85), and all-cause mortality or readmission (RR = 0.71, 95% CI = 0.61–0.82). However, nurse-led heart failure self-care education was not associated with improvements in the quality of life and heart failure knowledge. Studies on the effectiveness of nurse-led heart failure self-care education mostly report only the positive effects on patients’ health outcomes, whereas evidence of the effectiveness of the nurse-led approach is still limited. Therefore, high quality randomized controlled trials with detailed and explicit descriptions on the components of the interventions are needed.

## 1. Introduction

Heart failure is a major public health concern that affects around 26 million people worldwide [1,2,3]. It has a high prevalence particularly among older adults. Patients aged 65 years or older have been reported to account for 80% and 90% of heart failure-related hospitalizations and deaths, respectively [4]. Despite advances in early diagnosis and treatment of heart failure, its progressive and debilitating characteristics [5] result in substantial morbidity and a high burden of long-term management, which negatively affects patients, families, and health systems [1,4,6]. According to previous studies, heart failure patients’ 30-day hospital readmission rate was about 20–30%, with up to 15% mortality after hospital discharge [7,8]. Health-related quality of life, a major outcome indicator of chronic health conditions, is known to be much worse in people with heart failure than those with other chronic conditions [5]. With the rapidly increasing number of older adults and the irreversible nature of heart failure, it poses an overwhelming burden requiring further efforts to reduce adverse health outcomes and improve quality of life.

Based on the guidelines reported by the European Society of Cardiology for the diagnosis and treatment of acute and chronic heart failure [9], adherence to self-care is an important component of improving patient outcomes—reducing mortality and improving the quality of life. Therefore, guidelines on heart failure management place a strong emphasis on adherence to self-care behaviors such as lifestyle modifications and fluid restrictions [10]. Recent reviews on the impact of heart failure self-management interventions highlight their beneficial effects in reducing rates of heart failure specific readmission and mortality, as well as improving heart failure-related quality of life [11,12]. However, previous studies have highlighted the problem of poor adherence to self-care in individuals with heart failure [13,14], which could be related to the complex nature of self-care, lack of perceived need for self-care, the burden of maintaining long-term behavioral changes, or a lack of motivation [14].

While transitioning from the hospital to the community, patients are expected to sustain their self-care knowledge and skills taught during hospitalization. Therefore, heart failure patients need self-care adherence to maintain hospital-initiated self-care education. Nurses are considered a crucial part of the heart failure health care system [15]. They play a pivotal role in providing patients with educational assistance by identifying access to expert information, promoting patients’ health literacy, and thus empowering them [16]. Nurse-led self-care interventions have many advantages such as patients’ favorable perception of nurses, high quality of intervention delivery, more frequent and periodic follow-ups, and potentially lower health care expenditure [17].

According to the results of a previous systematic review, nurse-led heart failure management programs reduced the heart failure specific readmission rate by 32% and all-cause readmission by 15% [18]. Considering that this review mainly focused on heart failure specific readmission rate as its main outcome [18], more evidence is required on the effects of nurse-led heart failure self-care education on comprehensive, patient-centered health outcomes, such as patient-reported quality of life or knowledge on heart failure. Nurses are a well-positioned but underused workforce that can lead self-care interventions and implement strategies to promote adherence in individuals with complex chronic conditions, such as heart failure. We defined nurse-led heart failure self-care education in which hospital nurses, regardless of delivery method, have a direct role in delivering the intervention to research participants for improving health outcomes after hospital discharge. They may operate alone or within a multidisciplinary team. Nurse-led heart failure self-care education should be considered as additional care in this systematic review. Therefore, we conducted a systematic review and meta-analysis of randomized controlled trials (RCTs) with the following aims: (1) to describe characteristics of the intervention, and (2) to examine the effects of nurse-led interventions on patients’ health outcomes.

## 2. Methods

### 2.1. Search Strategies

We used the guideline for the Preferred Reporting Items for Systematic Reviews and Meta-Analyses (PRISMA) [19] and devised research questions using the PICO format for systematic review [20]: for heart failure patients (Participants), is hospital-based, nurse-led heart failure self-care education (Intervention) more beneficial to the health outcomes (Outcomes) for intervention group than the control group (Comparison) in RCTs (Type of studies)? Before undertaking this systematic review, we checked whether there are already existing or ongoing reviews in the Cochrane library Joanna Briggs Institute and International prospective register of systematic review. Our review included only RCTs to reduce the certain sources of bias when testing the effectiveness of nurse-led heart failure self-care education. To confirm all relevant articles published between January 2000 and October 2019, we conducted a systematic search of six databases: PubMed, EMBASE, Cochrane library, CINAHL, Web of Science, and SCOPUS. The following search terms based on Medical Subject Heading (MeSH) terms and keywords were used: (“heart failure” OR “cardiac failure” OR “congestive heart failure” OR “heart decompensation” OR “myocardial failure”) AND (“self-care” OR “self-care behavior” OR “self-care behavior” OR “self-management) AND (“nurse-led” OR “nursing-led). Additional studies were identified by reviewing references lists of already identified studies (Table S1).

### 2.2. Study Selection 

This review included studies meeting the following criteria: (1) original articles on RCTs that tested interventions on heart failure self-care or self-management that are designed and/or delivered by nurses, (2) published in English, and (3) comprising patients aged 18 years or older receiving treatment for a diagnosis of heart failure. It excluded: (1) editorials, observational studies, letters, conference abstracts, and reviews; (2) studies with no outcomes; and (3) studies without peer reviews.

**Figure 1:**
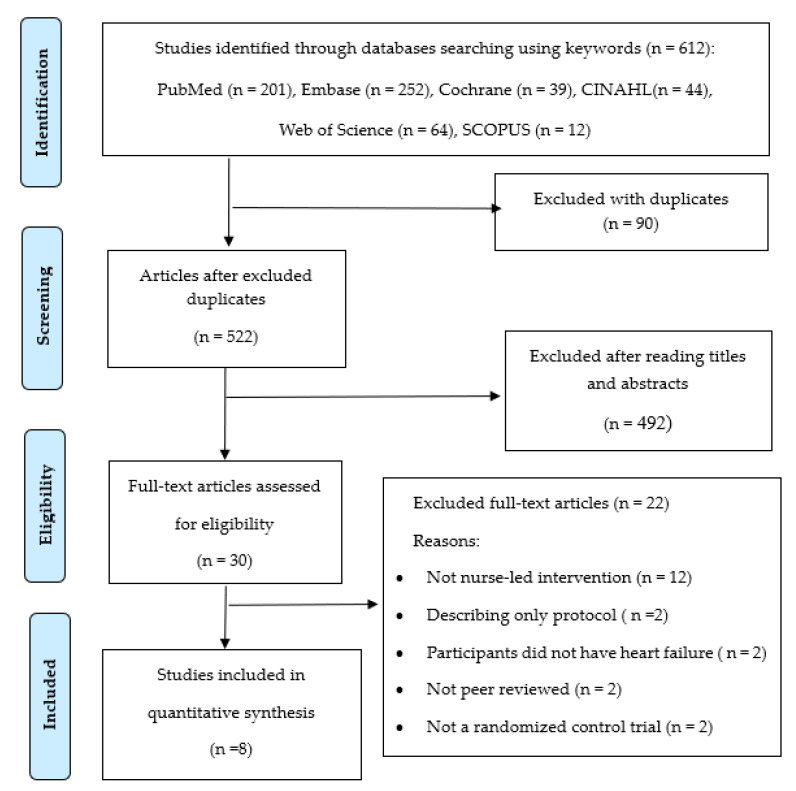
Flow chart of systematic review of literature.

Regarding health outcomes, the primary outcomes were all-cause readmission, heart failure specific readmission, and all-cause mortality, whereas the secondary outcomes included patient-reported quality of life and heart failure knowledge. To ensure reliability, two reviewers (YJS and HJL) independently selected relevant articles by screening retrieved titles and abstracts. Any discrepancies were resolved through discussion. Finally, eight RCTs were identified and included in this analysis. The total process and the exclusion causes are shown in Figure 1. 

### 2.3. Data Extraction

Data on outcomes were elicited from each study using a data extraction sheet organized by: first author’s last name, publication year, study location, patient characteristics (e.g., sample size, mean age, percentage of males and females, and severity of heart failure), initiation of the intervention, intervention length, intervention group details, control group details, outcome variables, and main findings (Table 1). Two authors (YJS and HJL) finished the data extraction independently and the third author (JYC) resolved disagreements.

### 2.4. Assessment of Methodological Quality

Two authors (YJS and HJL) independently evaluated the quality of the studies included in this review, and their level of agreement was recorded. Any disagreements between the two authors were resolved through discussion with a third author (JYC). The quality of each study was assessed as high, low, or unclear using the Cochrane Risk of Bias (RoB) tool [21].

### 2.5. Data Synthesis

We performed a meta-analysis using Comprehensive Meta-analysis software (version 3.0; Biostat, Englewood, NJ, USA) to determine the differences between the intervention and the standard care groups in their risks of health outcomes. Individual effect sizes were calculated as risk ratio (RR) with 95% confidence intervals (CI). We used the random-effects model considering the heterogeneity of the study design (e.g., sample size and duration of intervention) [22]. The I^2^ statistic with its 95% CI and X^2^ test (statistical significance when *p* < 0.05) were used to measure heterogeneity. Since the meta-analysis results included less than 10 studies, we were unable to evaluate the publication bias [23].

## 3. Results

### 3.1. Study Quality Appraisal

**Figure 2:**
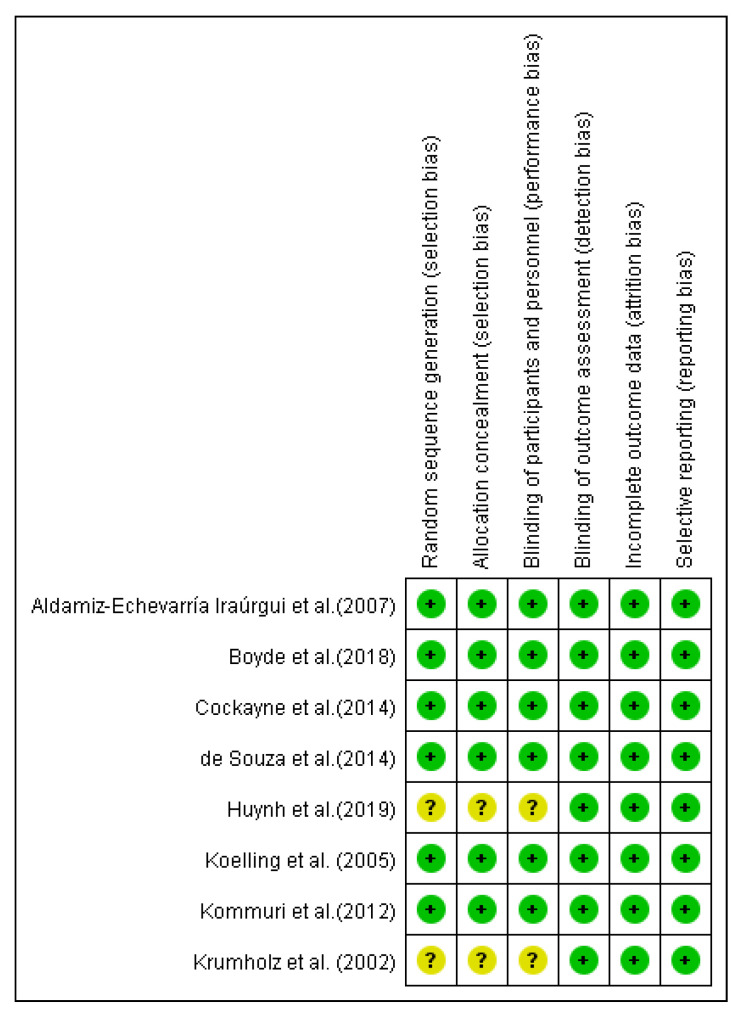
Risk of bias graph.

**Figure 3:**
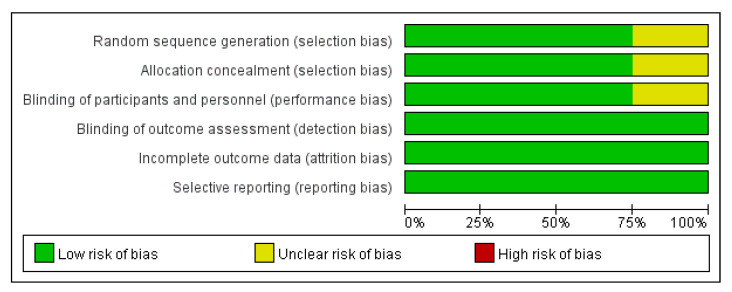
Risk of bias summary.

This review was judged as having a relatively low overall risk of bias (Figure 2). The three domains of detection, attrition, and reporting were generally adequate in all the eight studies. Random sequence generation, allocation concealment, and blinding of data collection were rated to be sufficient strategies in six studies (75%), but these domains were not addressed in two studies [24,31]. Therefore, the risks of bias were not clear in these studies (Figure 3). 

### 3.2. Study Settings and Patient Characteristics Included

As shown in Table 1, most studies were conducted in Western countries such as the USA, Europe, and Australia. A total of 1979 patients were covered in this systematic review, of which 949 and 1030 were assigned to the intervention and control groups, respectively. Among the patients, 1167 (59%) were men and 812 (41%) were women. Their mean age ranged from 64 to 76 years. They were recruited regardless of time elapsed since the diagnosis of heart failure. In approximately 71% of heart failure patients, the mean left ventricular ejection fraction (LVEF) was less than 40%.

### 3.3. Components of Heart Failure Self-Care Education Interventions

These were classified as: single-component (*n* = 3) and multicomponent (*n* = 5). Three single component studies reported only educational interventions [25,27,30], with a one-day delivery duration. The time of initiating self-care education was prior to discharge, but this information was not reported in one study [30]. The follow-up periods were: 3, 6, and 12 months. All the interventions were provided once in the hospital setting, and their contents mainly comprised basic facts about heart failure and its management (i.e., symptoms, lifestyle, diet, and therapy). While all studies used one-to-one discussions as the modality of intervention delivery, one study added video viewing as multimedia education [30]. The studies’ educational materials included information booklets, manuals, guidelines, and DVDs (Table 1). 

Five studies were introduced as multi-component nurse-led self-care education interventions [24,26,28,29,31]. In this review, multi-component self-care education was defined as adding additional strategies such as additional phone calls, clinical assessments, and home visits to promote education. Self-care education combined with phone calls or home visits were the most frequently used strategies in the included studies. One study provided four components: self-care education, phone calls, home visits, and clinical assessment [31]. The duration of intervention varied from 2 weeks to 12 months, and its initiation was categorized as: prior to discharge or within 10~15 days after discharge. The number of intervention sessions offered in each study ranged from 4 to 17. The follow-up periods were categorized as 3, 6, and 12 months (Table 1).

### 3.4. Study Outcomes

**Figure 4:**
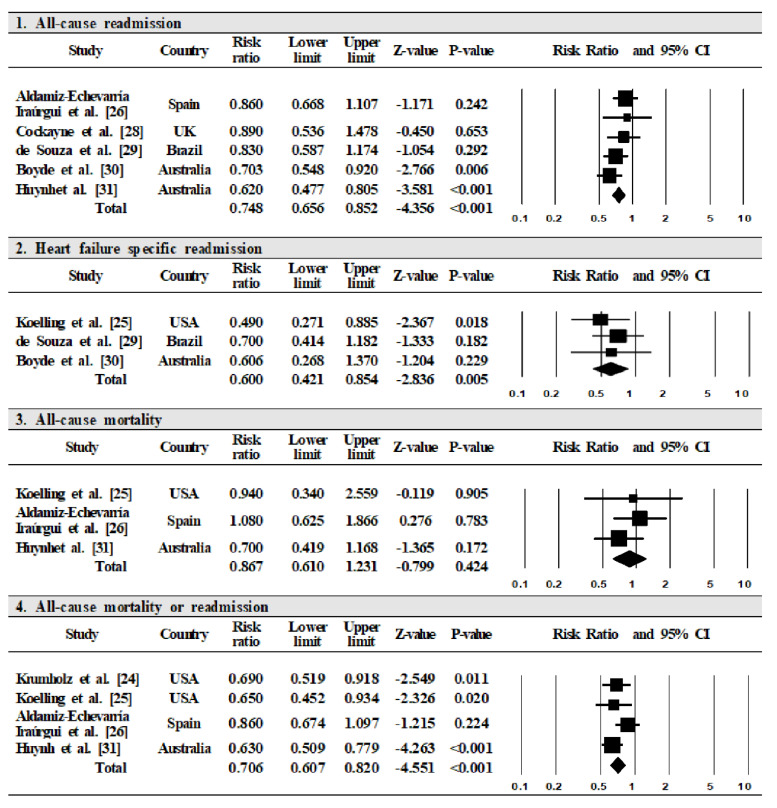
The effect of nurse-led self-care education intervention on health outcomes. Note: CI = confidence interval.

The primary outcomes included were: all-cause readmission, heart failure specific readmission, and all-cause mortality. First, five studies examined data on all-cause readmission [26,28,29,30,31]. The meta-analysis demonstrated a 25.2% reduction in the risk of all-cause readmission in the intervention group compared to the control group (RR = 0.748, 95% CI = 0.656–0.852). The pooled results confirmed low heterogeneity (I^2^ = 4%, *p* = 0.382) (Figure 4). Second, three studies examined data on heart failure specific readmission [25,29,30]. The meta-analysis demonstrated a 40.0% reduction in the risk of heart failure specific readmission in the intervention group compared to the control group (RR = 0.600, 95% CI = 0.421–0.854). The pooled results confirmed low heterogeneity (I^2^ = 0%, *p* = 0.676) (Figure 4). Third, three studies examined data on all-cause mortality [25,26,31]. The meta-analysis demonstrated a 13.3% reduction in the risk of all-cause mortality for the intervention group compared to the control group (RR = 0.867, 95% CI = 0.610–1.231), but the result was not statistically significant. The pooled results confirmed low heterogeneity (I^2^ = 0%, *p* = 0.518) (Figure 4). Lastly, four studies examined data on all-cause mortality or readmission [24,25,26,31]. The meta-analysis demonstrated a 29.4% reduction in the risk of all-cause mortality or readmission in the intervention group compared to the control group (RR = 0.706, 95% CI = 0.607 to 0.820) (Figure 4). The pooled results confirmed low heterogeneity (I^2^ = 22%, *p* = 0.278).

With regard to secondary outcomes, two studies showed that nurse-led heart failure self-care education interventions had no significant effect on heart failure patients’ quality of life [25,28]. The results of the studies that investigated heart failure knowledge as the effect of nurse-led heart failure self-care education were inconsistent, with two studies reporting significant improvements in heart failure knowledge [27,29], whereas the other study demonstrated no significant effect [30].

## 4. Discussion

In this systematic review, we examined the effects of nurse-led heart failure self-care education on patients’ health outcomes and further explored the features of its modalities. The results of random-effects meta-analysis revealed that nurse-led heart failure self-care education reduced heart failure patients’ all-cause readmission by 25.2%, heart failure specific readmission by 40.0%, and all-cause mortality or readmission by 29.4%. Although all-cause mortality reduced by 13.3%, the change was not statistically significant.

Results of the current review demonstrate that nurse-led heart failure self-care education was effective in reducing all-cause readmission and particularly heart failure specific readmission. Nurses are more suitable than other professionals as primary educators of home care for patients discharged with chronic illness because they can build trusting relationships by spending more time communicating with patients and their families [32,33]. Trust in relationships between nurses and patients can help in enhancing adherence to recommended self-care behaviors, identifying errors in the discharge plan to reduce unexpected readmission after discharge, as well as in developing a thorough knowledge of the patient’s individual discharge needs [34]. Additionally, nurses have access to expertise as needed and can be assisted by other clinical disciplines. In this review, nurses promoted self-care for discharged heart failure patients through heart failure knowledge assessment, physical examinations, psychosocial support, and education on heart failure management. Our findings were consistent with previous reviews that reported the effects of nurse-led heart failure self-care education on promoting healthy behaviors [35,36]. Studies have also demonstrated the effects of health care professionals’ heart failure management education in other disciplines. For example, engaging pharmacists in heart failure management demonstrated improved medication adherence [37], and dietitian-administered counseling was effective in adherence to low-sodium diets [38]. While interventions in previous studies were limited to the focus corresponding to disciplines such as diet or medication, nurse-led self-care interventions have the advantage of providing further integrated heart failure management and could, therefore, be more favorable for improving clinical outcomes and promoting self-care behaviors in heart failure patients.

However, in the current review, the nurse-led heart failure self-care education may have not demonstrated a significant reduction in all-cause mortality partly because of the shorter intervention period. Three of the studies used for meta-analysis had intervention periods ranging from 1 or 15 days [25,26,31]. Since heart failure is a chronic condition that involves progressive deterioration over time, living with it requires good self-care through complex long-term management, such as sodium restrictions, symptom monitoring, adherence to medical treatment, and promoting physical activity and exercise [39]. Accordingly, heart failure patients may find it challenging to process large amounts of new information if the nurse-led heart failure self-care education is provided for a short period of time. Modifying the intervention period could, therefore, be a way to improve nurse-led heart failure self-care education and its effects on decreasing mortality. We also speculate the focus of the intervention might not be well aligned with patients’ real life goals and priorities of self-care. So participant engagement might not be sufficient enough to draw desired outcomes. Another explanation is that the mortality estimated in this meta-analysis was all-cause mortality, because it included studies that had enrolled patients with various comorbidities (e.g., atrial fibrillation, chronic obstructive pulmonary disease, chronic kidney disease, diabetes) and heart failure may have been just one of the multiple conditions that contributed to mortality. Previous studies have identified that heart failure patients with comorbidities have a poor survival rate [40,41]. In this context, support for managing comorbidities is essential in interventions for improving heart failure patients’ self-care abilities. Further research is, therefore, necessary to identify the effects of nurse-led heart failure self-care education on heart failure-cause mortality.

The results of our systematic review showed that the effects of nurse-led heart failure self-care education on quality of life were not significant [25,28]. This is probably because quality of life is a representative measure of life satisfaction and is not considered a suitable outcome measure for a short-term intervention [42]. Therefore, the two studies in this review that included one or a few educational sessions and follow-up at short-term intervals may have been inadequate for improving the quality of life. This result indicates that nurse-led heart failure self-care education can be largely determined by the education period and frequency along with extended follow-up intervals to examine long-term outcomes. Therefore, more studies are needed to decide upon the most optimal intervention period and the frequency to evaluate quality of life in this context. We are also unable to draw a conclusion regarding the effect of nurse-led heart failure self-care education on heart failure knowledge because of the inconsistency in results between two studies. The study that evaluated heart failure knowledge using the Dutch Heart Failure Knowledge Scale (DHFKS) reported no significant effects [30], whereas, the other study that used a Heart Failure Knowledge Questionnaire (HFKQ) developed by the study team reported significant results [27]. While both studies measured the same heart failure knowledge, they used different instruments. While the DHFKS has been presented as a reliable and valid instrument for evaluating heart failure knowledge [43], the psychometric properties of the HFKQ have not yet been evaluated. Therefore, future researchers should provide reliable evidence by using international and standardized instruments for benchmarking the results. 

It is important to include patients as well as their family members in heart failure management education [44]. The Joint Commission stipulates the inclusion of family caregivers in the discharge education process [45]. In addition to physical problems, psychological maladjustment and lack of social support could impede older patients in conducting routine activities of daily living [46]. According to a recent review, heart failure dyadic self-care intervention is associated with better cognitive, behavioral, affective, and health services utilization outcomes [47]. In this review, the heart failure patients’ age range was 62–76 years. Although this indicates the need for family participation in discharge education, only two of the eight studies had included relatives in discharge education [26,29]. For improving health outcomes of discharged heart failure patients, future studies should consider their age and include their families in discharge education. In particular, focused self-care education is imperative for older heart failure patients without family members, who face a greater likelihood of social isolation after discharge and are highly vulnerable to poor self-care behaviors.

Importantly, we found that outcome variables were not significant in three studies that provided nurse-led heart failure self-care education after hospital discharge [26,28,29]. Previous studies highlighted that the absence of patient education before discharge and deficiency of preparedness for discharge were predictors of adverse health outcomes [48]. If patients are not well-informed about managing heart failure at home before discharge, it could lead to clinical deterioration. Pre-discharge interventions should be developed as a part of discharge planning and should involve assessment of the patients’ knowledge and lifestyle [49]. Lambrinou et al. identified that heart failure management programs provided before discharge were effective in improving health outcomes [18]. Therefore, prior to discharge, health care professionals should initiate interventions such as a discharge education session so that discharged patients can have effective heart failure management at home.

Although heart failure is a global health issue [2,50], most studies identified in this review were conducted in high-income countries, (the exception being Brazil, a middle-income country) [29], where the impact of nurse-led heart failure self-care education has been proven in terms of reducing readmission and mortality. Therefore, future research should investigate the effects of nurse-led heart failure self-care education through global comparisons reflecting diversity in economic resources and culture. Furthermore, this review had limited information on nurses’ qualifications, preparedness, or their specific responsibilities in delivering nurse-led education interventions. Only one study reported the work experience and educational qualifications of nurses participating in nurse-led interventions [30]. Consequently, future studies need to describe the role and responsibilities of nurses in conducting nurse-led heart failure interventions. The contents of education in the individual studies in this review are very diverse and different, so it was not possible to accurately clarify what standardized education or format of delivery for patients should receive. Further research should describe the contents of standardized education or format of delivery for heart failure patients.

There are several significant limitations. First, the small sample size and limited number of studies included in this meta-analysis limited generalizability. Second, patient satisfaction was not synthesized because it was not reported in the included studies. Patient satisfaction forms part of patient-reported outcomes and could be a significant indicator of successful intervention delivery. Third, none of the studies reviewed provided descriptions of the intervention in sufficient detail. Further RCTs that would suggest full description of study protocol, and contents or scope of self-care behaviors are, therefore, needed. Finally, since we did not include articles published in languages other than English, there is the likelihood of this review having overlooked relevant research published in other languages. 

## 5. Conclusions

Our findings highlight the positive effects of nurse-led heart failure self-care education on clinical outcomes such as readmission and mortality. However, it is not clear whether nurse-led interventions are effective in patient reported quality indicators, including quality of life and disease knowledge. Thus, there is a need for more RCTs with longer follow-ups that can have long-term effects and influence on patients’ behavioral changes. Moreover, nurse-led heart failure education programs should be systematically planned prior to hospital discharge, as well as at the time at diagnosis. For improving the effectiveness of nurse-led approaches, the role and responsibilities of nurses in conducting nurse-led heart failure interventions should be explicitly described in each program. Nurse-led education programs should be planned prior to hospital discharge, as well as at the time at diagnosis.

## Figures and Tables

**Table 1:** Description of studies included (*N* = 8).

Authors (Publication Year)/Location	Participants			Time of Initiation	Duration of Intervention Delivery	Follow-Up Period	Intervention	Outcome Variables	Main Findings
	Intervention Group	Control Group							
Krumholz et al. (2002)/USA [24]	*N* = 44 Mean age: 75.9 yr M: 48%, F: 52% NYHA: unreported Mean LVEF: 38.0%	*N* = 44 Mean age: 71.6 yr M: 66%, F: 34% NYHA: unreported Mean LVEF: 37.0%		Prior to discharge	12 months	12 months	·Intervention: Cardiac nurse reviewed (once/1 h) patient’s knowledge of heart failure in one to one and used phone call (17 times) to support heart failure care and management. ·Control: Not detail reported.	All-cause mortality or readmission, heart failure mortality or readmission, costs	Intervention group had significantly lower on all-cause mortality or hospital readmission (*p* = 0.01), heart failure mortality or readmission (*p* = 0.01), costs (*p* = 0.02)
Koelling et al. (2005)/USA [25]	*N* = 107 Mean age: 65 yr M: 58%, F: 42% NYHA: unknown Mean LVEF: 26%	*N* = 116 Mean age: 64 yr M: 58%, F: 42% NYHA: unknown Mean LVEF: 27%		Prior to discharge	1 day	6 months	·Intervention: Nurse educator discussed (once/1 h) heart failure-specific information that covered the basic principles of the causes of heart failure and rationale for pharmaceutical therapies. ·Control: Received standard written discharge information.	All-cause mortality or readmission, all-cause mortality, heart failure specific readmission, cost	Intervention group had significantly lower on all-cause mortality or readmission (*p* = 0.018), heart failure specific readmission (*p* = 0.015), cost (*p* = 0.035); No significant difference in all-cause mortality, QoL
Aldamiz-Echevarría Iraúrgui et al. (2007)/Spain [26]	*N* = 137 Mean age: 75 yr M: 39%, F: 61% NYHA: unreported Mean LVEF: 50.9%	*N* = 142 Mean age: 76 yr M: 40%, F: 60% NYHA: unreported Mean LVEF: 48.3%		Within 15 days after hospital discharge	15 days	12 months	·Intervention: Nurse visited home 3 times (1 h) after discharge to administer the education program, teaching patient and relatives basic facts about heart failure and its management (symptoms, lifestyle, diet, therapy) ·Control: Not detail reported.	All-cause mortality or readmission, all-cause mortality, all-cause readmission	No significant difference in all-cause mortality or readmission, all-cause mortality, all-cause readmission
Kommuri et al. (2012)/USA [27]	*N* = 128 Mean age: 66 yr M: 61%, F: 39% NYHA: unknown Mean LVEF: 25%	*N* = 137 Mean age: 67 yr M: 61%, F: 39% NYHA: unknown Mean LVEF: 26.5%		Prior to discharge	1 day	3 months	·Intervention: Nurse educated (once/1 h) the basic principles of heart failure, role of dietary sodium, importance of limitation of fluid intake, the mechanisms of diuretics, and the rationale for other pharmacotherapy. ·Control: Received standard discharge information.	Heart failure knowledge	Intervention group had significantly higher on heart failure knowledge (*p* = 0.007).
Cockayne et al.(2014) /UK [28]	*N* = 95 Mean age: 70 yr M: 73%, F: 27% NYHA: Ⅰ-Ⅱ= 73%, Ⅲ-Ⅳ= 27%, Mean LVEF: unreported	*N* = 165 Mean age: 70 yr M: 72%, F: 28% NYHA: Ⅰ-Ⅱ= 66%, Ⅲ-Ⅳ= 34%, Mean LVEF: unreported		After discharge	12 months	12 months	·Intervention: Specialist heart failure nurse provided (up to 6 times) self-management program (The heart plan) and accompanying DVD, relaxation tape, exercises, regular monitoring of symptoms, blood tests, clinical assessments, referrals.·Control: Given the same self-management manual.	All-cause readmission, QoL	No significant difference in all-cause readmission, QoL
de Souza et al.(2014)/ Brazil [29]	*N* = 123 Mean age: 62 yr M: 61 %, F: 39% NYHA: Ⅰ-Ⅱ= 46%, Ⅲ-Ⅳ= 54%, Mean LVEF: 29%	*N* = 129 Mean age: 62 yr M: 64%, F: 36% NYHA: Ⅰ-Ⅱ= 42%, Ⅲ-Ⅳ= 58%, Mean LVEF: 30%		Within 10 days after hospital discharge	4 months	6 months	·Intervention: Nurses provided a heart failure-focused physical examination (Clinical Congestion Score, blood pressure, jugular venous pressure) through a home visits (4 times/~60 min). Phone calls (4 times/~10 min) were used to reinforce recommendations given during home visits, check the use of prescribed medications. ·Control: Received instructions regarding pharmacological and non-pharmacological therapeutic strategies.	All-cause mortality, all-cause readmission, heart failure specific readmission & ED visits, heart failure knowledge	Intervention group had significantly higher on heart failure knowledge (*p* < 0.001); No significant difference in all-cause mortality, all-cause readmission, heart failure specific readmission & ED visits
Boyde et al. (2018)/ Australia [30]	*N* = 100 Mean age: 64 yr M: 77%, F: 23% NYHA: Ⅰ-Ⅱ= 35%, Ⅲ-Ⅳ= 65%, LVEF: <36%: 75%	*N* = 100 Mean age: 64 yr M: 69%, F: 31% NYHA: Ⅰ-Ⅱ= 31%, Ⅲ-Ⅳ= 69%, LVEF: <36%: 83%		Unreported	1 day	12 months	·Intervention: Patients watched the DVD for 30 min on the role model of self-care behaviors and participated in a one to one discussion with a specialist heart failure nurse (once/60~90 min) ·Control: Received 30–60 min of standard education and offered a short booklet outlining a brief overview of diagnosis, symptoms, and treatment of heart failure.	All-cause readmission, heart failure specific readmission, heart failure knowledge, self-care behaviors	Intervention group had significantly lower on all-cause readmission (*p* = 0.005); No significant difference in heart failure specific readmission, heart failure knowledge, self-care behaviors
Huynh et al. (2019)/ Australia [31]	*N* = 215 Mean age: 73.9 yr M: 51%, F: 49% NYHA: Ⅰ–Ⅱ = 30%, Ⅲ–Ⅳ = 70%, Mean LVEF: 39%	*N* = 197 Mean age: 74.7 yr M: 58%, F: 42% NYHA: Ⅰ–Ⅱ = 35%, Ⅲ–Ⅳ = 65%, Mean LVEF: 40%	Prior to discharge	2 weeks	3 months	·Intervention: Patients were checked for intravascular capacity before discharge and educated in self-care and exercise by leaflet and video instruction. Cardiac nurse’s phone calls (twice) for transition coach and support. Home visits (twice) provided an opportunity to react to any outstanding or emerging issues to prevent them from growing into more serious events, as well as to provide patients with mental and physical support. Control: Received a standard disease management program and standard discharge plan.		All-cause mortality or readmission, all-cause mortality, all-cause readmission	Intervention group had significantly lower on all-cause mortality or readmission (95% CI = 0.46–0.84), all-cause readmission (95% CI = 0.45–0.88); No significant difference in all-cause mortality

Note: IG = intervention group; CG = control group; M = male; F = female; NYHA = New York Heart Association; LVEF = left ventricular ejection fraction; QoL = quality of life; CD-ROM = compact disc read only memory; ED = emergency department; DVD = digital versatile disc; CI = confidence interval.

## Supplementary Materials

The following are available online at https://www.mdpi.com/1660-4601/17/18/6559/s1, Table S1: Search results.
