# Supplementary material for: Effectiveness of Nurse-Led Heart Failure Self-Care Education on Health Outcomes of Heart Failure Patients: A Systematic Review and Meta-Analysis
Source: Int J Environ Res Public Health. 2020 Sep 9;17(18):6559. doi: 10.3390/ijerph17186559 (PMC7560014; doi:10.3390/ijerph17186559)
Supplement: Supplementary file 1 [file ijerph-17-06559-s001.pdf]

Table S1. Search Results.

Database	Number	Search query	Results
PubMed	#1	("Heart Failure"[mesh] OR "Heart Failure"[tiab] OR "Cardiac Failure"[tiab] OR "Heart Decompensation"[tiab] OR "Myocardial Failure"[tiab] OR "Congestive Heart Failure"[tiab] OR HF[tiab] OR "Chronic heart failure"[tiab] OR "heart failure, congestive"[tiab] OR "systolic dysfunction"[tiab])	227,131
	#2	"Hospitalization"[Mesh] OR Hospitalization[tiab] OR "Patient Admission"[Mesh] OR "Patient Admission"[tiab] OR admission[tiab]	415,931
	#3	"Patient Discharge"[Mesh] OR "Patient Discharge"[tiab] OR "Patient Discharge Summaries"[Mesh] OR "Patient Discharge Summar*"[tiab] OR "Patient Education as Topic"[Mesh] OR "Patient Education Handout" [Publication Type] OR "Patient Education"[tiab] OR "Discharge Education"[tiab]	123,744
	#4	"Self Care"[Mesh] OR "Self-care"[tiab] OR "Self-care behaviors"[tiab] OR "Hospitalization"[Mesh] OR Hospitalization[tiab] OR Rehospitalization*[tiab] OR "Patient Readmission"[Mesh] OR Readmission[tiab] OR "Mortality"[Mesh] OR "mortality" [Subheading] OR Mortality[tiab] OR "Death"[Mesh] OR death[tiab]	2,010,352
	#5	"randomized controlled trial"[Publication Type] OR "randomized controlled trials as topic"[MeSH] OR "clinical trial"[Publication Type] OR "clinical trials as topic"[MeSH] OR "Non-Randomized Controlled Trials as Topic"[Mesh] OR "Randomized Controlled Trial"[tiab] OR RCT[tiab] OR "Clinical trial"[tiab] OR Program[tiab] OR Intervention[tiab] OR Management[tiab] OR Experimental[tiab] OR "Quasi-experimental"[tiab]	3,846,984
	#6	#1 AND #2 AND #3 AND #4 AND #5	723
	#7	#**6** AND ((Clinical Trial[ptyp] OR Controlled Clinical Trial[ptyp] OR Pragmatic Clinical Trial[ptyp] OR Randomized Controlled Trial[ptyp]) AND ("0001/01/01"[PDAT] : "2019/07/31"[PDAT]) AND "humans"[MeSH Terms] AND English[lang])	**201**
EMBASE	#1	heart failure'/de OR 'backward failure, heart':ti,ab OR 'cardiac backward failure':ti,ab OR 'cardiac decompensation':ti,ab OR 'cardiac failure':ti,ab OR 'cardiac incompetence':ti,ab OR 'cardiac insufficiency':ti,ab OR 'cardiac stand still':ti,ab OR 'cardial decompensation':ti,ab OR 'cardial insufficiency':ti,ab OR 'chronic heart failure':ti,ab OR 'chronic heart insufficiency':ti,ab OR 'decompensatio cordis':ti,ab OR 'decompensation, heart':ti,ab OR 'heart backward failure':ti,ab OR 'heart decompensation':ti,ab OR 'heart failure':ti,ab OR 'heart incompetence':ti,ab OR 'heart insufficiency':ti,ab OR 'insufficientia cardis':ti,ab OR 'myocardial failure':ti,ab OR 'myocardial insufficiency':ti,ab OR 'congestive heart	411,265

		failure'/de OR 'cardiac congestive failure':ti,ab OR 'congestive cardiac failure':ti,ab OR 'congestive heart failure':ti,ab OR 'congestive heart insufficiency':ti,ab OR 'heart failure, congestive':ti,ab OR hf:ti,ab OR 'systolic dysfunction'/de OR 'systolic dysfunction':ti,ab	
	#2	hospitalizationion:ti,ab OR 'hospital admission'/de OR 'admission, hospital':ti,ab OR 'admitting department, hospital':ti,ab OR 'hospital admission':ti,ab OR 'hospital admittance':ti,ab OR 'patient admission':ti,ab OR 'waiting list':ti,ab OR 'waiting lists':ti,ab OR 'admission'/de OR 'admissions'/de	200,304
	#3	hospital discharge'/de OR 'discharge planning':ti,ab OR 'discharge, hospital':ti,ab OR 'hospital discharge':ti,ab OR 'patient discharge':ti,ab OR 'medical record'/de OR 'dental record':ti,ab OR 'dental records':ti,ab OR 'diet records':ti,ab OR 'forms and records control':ti,ab OR 'health records, personal':ti,ab OR 'history, morbid':ti,ab OR 'hospital record':ti,ab OR 'hospital records':ti,ab OR 'medical archive':ti,ab OR 'medical data storage':ti,ab OR 'medical record':ti,ab OR 'medical record linkage':ti,ab OR 'medical records':ti,ab OR 'medical records department, hospital':ti,ab OR 'medical records, problem-oriented':ti,ab OR 'medical secret':ti,ab OR 'medical transcription':ti,ab OR 'morbid history':ti,ab OR 'morbid record':ti,ab OR 'nursing records':ti,ab OR 'patient discharge summaries':ti,ab OR 'patient discharge summary':ti,ab OR 'patient generated health data':ti,ab OR 'patient portals':ti,ab OR 'patient record':ti,ab OR 'psychiatric record':ti,ab OR 'record, dental':ti,ab OR 'record, medical':ti,ab OR 'patient education'/de OR 'education, patient':ti,ab OR 'patient education':ti,ab OR 'patient education as topic':ti,ab OR 'patient medication knowledge':ti,ab OR 'discharge education':ti,ab	502,716
**EMBASE**	#4	self care'/de OR 'self care':ti,ab OR 'self management':ti,ab OR 'self treatment':ti,ab OR 'self-management':ti,ab OR 'selfcare':ti,ab OR 'selfmanagement':ti,ab OR 'selftreatment':ti,ab OR 'self-care behaviors':ti,ab OR 'hospitalization'/de OR 'hospital stay':ti,ab OR 'hospitalization':ti,ab OR 'short stay hospitalization':ti,ab OR rehospitalization*:ti,ab OR 'hospital readmission'/de OR 'hospital readmission':ti,ab OR 'patient readmission':ti,ab OR 'readmission':ti,ab OR 'readmission rate':ti,ab OR 'readmissions':ti,ab OR 'rehospitalization':ti,ab OR 'mortality'/de OR 'excess mortality':ti,ab OR 'mortality':ti,ab OR 'mortality model':ti,ab OR 'death'/de OR 'death':ti,ab OR 'mors':ti,ab	2,437,685
	#5	randomized controlled trial'/de OR 'controlled trial, randomized':ti,ab OR 'randomised controlled study':ti,ab OR 'randomised controlled trial':ti,ab OR 'randomized controlled study':ti,ab OR 'randomized controlled trial':ti,ab OR 'trial, randomized controlled':ti,ab OR 'randomized	4,850,896

		controlled trial (topic)'/de OR 'pragmatic clinical trials as topic':ti,ab OR 'randomized controlled trial (topic)':ti,ab OR 'randomized controlled trials':ti,ab OR 'randomized controlled trials as topic':ti,ab OR 'clinical trial'/de OR 'clinical drug trial':ti,ab OR 'clinical trial':ti,ab OR 'major clinical trial':ti,ab OR 'trial, clinical':ti,ab OR program:ti,ab OR 'intervention study'/de OR 'intervention studies':ti,ab OR 'intervention study':ti,ab OR 'intervention trial':ti,ab OR 'interventional studies':ti,ab OR 'interventional study':ti,ab OR 'interventional trial':ti,ab OR intervention:ti,ab OR 'management'/de OR 'institutional management teams':ti,ab OR 'management':ti,ab OR 'management audit':ti,ab OR 'office management':ti,ab OR 'pharmacy administration':ti,ab OR 'policy making':ti,ab OR 'practice management':ti,ab OR 'practice management, dental':ti,ab OR 'practice management, medical':ti,ab OR 'practice management, veterinary':ti,ab OR 'practice valuation and purchase':ti,ab OR experimental:ti,ab OR 'quasi experimental study'/de OR 'quasi experimental study':ti,ab OR 'quasiexperimental study':ti,ab OR 'quasi experimental':ti,ab	
	#6	#1 AND #2 AND #3 AND #4 AND #5	666
	#7	**#6 AND ([article]/lim OR [article in press]/lim) AND [english]/lim AND [humans]/lim AND ('clinical trial'/de OR 'controlled clinical trial'/de OR 'controlled study'/de OR 'intervention study'/de OR 'randomized controlled trial'/de OR 'randomized controlled trial (topic)'/de OR 'total quality management'/de)**	**252**
**CINAHL**	#1	(MM "Heart Failure+")	27,853
	#2	TI ("Heart Failure" OR "Cardiac Failure" OR "Heart Decompensation" OR "Myocardial Failure" OR "Congestive Heart Failure" OR HF OR "Chronic heart failure" OR "heart failure, congestive" OR "systolic dysfunction") OR AB ("Heart Failure" OR "Cardiac Failure" OR "Heart Decompensation" OR "Myocardial Failure" OR "Congestive Heart Failure" OR HF OR "Chronic heart failure" OR "heart failure, congestive" OR "systolic dysfunction")	48,183
	#3	S1 OR S2	53,693
	#4	(MM "Hospitalization+")	19,723
	#5	(MM "Patient Admission")	6,885
	#6	TI (Hospitalizationion OR "Patient Admission" OR admission) OR AB (Hospitalizationion OR "Patient Admission" OR admission)	61,810
	#7	S4 OR S5 OR S6	81,259
	#8	(MM "Patient Discharge+")	13,276
	#9	(MM "Patient Discharge Summaries")	72
	#10	(MM "Patient Education+")	29,989
	#11	(MM "Patient Discharge Education")	836
	#12	TI ("Patient Discharge" OR "Patient Discharge Summar*" OR "Patient Education" OR "Discharge Education") OR AB	10,759

		("Patient Discharge" OR "Patient Discharge Summar*" OR "Patient Education" OR "Discharge Education")	
	#13	S8 OR S9 OR S10 OR S11 OR S12	48,680
	#14	(MM "Self Care+")	23,785
	#15	(MM "Hospitalization+")	19,723
	#16	(MM "Readmission")	5,588
	#17	(MM "Mortality+")	26,448
	#18	(MM "Death+")	24,192
	#19	TI ("Self-care" OR "Self-care behaviors" OR Hospitalization OR Rehospitalizations OR Readmission OR Mortality OR death) OR AB ("Self-care" OR "Self-care behaviors" OR Hospitalization OR Rehospitalizations OR Readmission OR Mortality OR death)	311,892
	#20	S14 OR S15 OR S16 OR S17 OR S18 OR S19	360,253
	#21	(MM "Randomized Controlled Trials+")	1,214
	#22	(MM "Clinical Trials+")	18,746
**CINAHL**	#23	(MM "Management+")	528,189
	#24	(MM "Experimental Studies+")	19,429
	#25	(MM "Quasi-Experimental Studies+")	132
	#26	TI ("Randomized Controlled Trial" OR RCT OR "Clinical trial*" OR Program* OR Intervention OR Management OR Experimental OR "Quasi-experimental") OR AB ("Randomized Controlled Trial" OR RCT OR "Clinical trial*" OR Program* OR Intervention OR Management OR Experimental OR "Quasi-experimental")	977,252
	#27	S21 OR S22 OR S23 OR S24 OR S25 OR S26	1,410,274
	#28	S3 AND S7 AND S13 AND S20 AND S27	61
	#29	#28 AND (제한자 - 출판년월일: -20190731; 영어; 인간)	**44**
	#1	MeSH descriptor: [Heart Failure] explode all trees	8,315
	#2	("Heart Failure" OR "Cardiac Failure" OR "Heart Decompensation" OR "Myocardial Failure" OR "Congestive Heart Failure" OR HF OR "Chronic heart failure" OR "heart failure, congestive" OR "systolic dysfunction"):ti,ab	26,468
**Cochrane**	#3	#1 OR #2	27,273
	#4	MeSH descriptor: [Patient Admission] explode all trees	569
	#5	(Hospitalizationion OR "Patient Admission" OR admission):ti,ab	21,719
	#6	#4 OR #5	21,999
	#7	MeSH descriptor: [Patient Discharge] explode all trees	1,361
	#8	MeSH descriptor: [Patient Discharge Summaries] explode all trees	10
	#9	MeSH descriptor: [Patient Education as Topic] explode all trees	8,338
**Cochrane**	#10	("Patient Discharge" OR "Patient Discharge Summar*" OR "Patient Education" OR "Discharge Education"):ti,ab	3,159
	#11	#7 OR #8 OR #9 OR #10	11,911
	#12	MeSH descriptor: [Self Care] explode all trees	5,304
	#13	MeSH descriptor: [Hospitalization] explode all trees	13,131
	#14	MeSH descriptor: [Mortality] explode all trees	12,756
	#15	MeSH descriptor: [Patient Readmission] explode all trees	956

	#16	MeSH descriptor: [Death] explode all trees	2,014
	#17	("Self-care" OR "Self-care behaviors" OR Hospitalization OR Rehospitalization* OR Readmission OR Mortality OR death):ti,ab	119,758
	#18	#12 OR #13 OR #14 OR #15 OR #16 OR #17	138,157
	#19	MeSH descriptor: [Randomized Controlled Trial] explode all trees	125
	#20	MeSH descriptor: [Clinical Trial] explode all trees	147
	#21	MeSH descriptor: [Programs] explode all trees	0
	#22	MeSH descriptor: [Non-Randomized Controlled Trials as Topic] explode all trees	50
	#23	("Randomized Controlled Trial" OR RCT OR "Clinical trial" OR Program* OR Intervention OR Management OR Experimental OR "Quasi-experimental"):ti,ab	575,507
	#24	#19 OR #20 OR #21 OR #22 OR #23	575,564
	#25	#3 AND #6 AND #11 AND #18 AND #24	**39**
**Web of Science**	#1	("Heart Failure" OR "Cardiac Failure" OR "Heart Decompensation" OR "Myocardial Failure" OR "Congestive Heart Failure" OR HF OR "Chronic heart failure" OR "heart failure, congestive" OR "systolic dysfunction") AND (Hospitalizationion OR "Patient Admission" OR admission) AND ("Patient Discharge" OR "Patient Discharge Summar*" OR "Patient Education" OR "Discharge Education") AND ("Self-care" OR "Self-care behaviors" OR Hospitalization OR Rehospitalizations OR Readmission OR Mortality OR death) AND ("Randomized Controlled Trial" OR "Randomised Controlled Trial" OR RCT OR "Clinical trial" OR Program OR Intervention OR Management OR Experimental OR "Quasi-experimental")	**64**
**SCOPUS**	#1	("Heart Failure" OR "Cardiac Failure" OR "Heart Decompensation" OR "Myocardial Failure" OR "Congestive Heart Failure" OR HF OR "Chronic heart failure" OR "heart failure, congestive" OR "systolic dysfunction") AND (Hospitalizationion OR "Patient Admission" OR admission) AND ("Patient Discharge" OR "Patient Discharge Summar*" OR "Patient Education" OR "Discharge Education") AND ("Self-care" OR "Self-care behaviors" OR Hospitalization OR Rehospitalizations OR Readmission OR Mortality OR death) AND ("Randomized Controlled Trial" OR "Randomised Controlled Trial" OR RCT OR "Clinical trial" OR Program OR Intervention OR Management OR Experimental OR "Quasi-experimental")	**12**
